# Tensile Deformation Behaviors of Pure Ti with Different Grain Sizes under Wide-Range of Strain Rate

**DOI:** 10.3390/ma16020529

**Published:** 2023-01-05

**Authors:** Misaki Deguchi, Shigeto Yamasaki, Masatoshi Mitsuhara, Hideharu Nakashima, Genki Tsukamoto, Tomonori Kunieda

**Affiliations:** 1 Interdisciplinary Graduate School of Engineering Sciences, Kyushu University, 6-1 Kasuga-kouen, Kasuga, Fukuoka 816-8580, Japan; 2 Department of Materials, Kyushu University, 744 Motooka, Nishi-ku, Fukuoka 819-0395, Japan; 3 Department of Advanced Materials Science and Engineering, Faculty of Engineering Sciences, Kyushu University, 6-1 Kasuga-kouen, Kasuga, Fukuoka 816-8580, Japan; 4 Steel Research Laboratories, Nippon Steel Corporation, 20-1 Shintomi, Futtsu, Chiba 293-0011, Japan

**Keywords:** CP-titanium, strain rates dependence, mechanical properties, twin, grain size, work hardening rate

## Abstract

In this study, pure titanium equivalent to Grade 1 was subjected to tensile tests at strain rates ranging from 10^−6^ to 10^0^ s^−1^ to investigate the relationship between its mechanical properties and its twinning and slip. Deformation properties and microstructures of samples having average grain sizes of 210 μm (Ti-210), 30 μm (Ti-30), and 5 μm (Ti-5) were evaluated. With increasing strain rates, the 0.2% proof stress and ultimate tensile strength increased for all samples; the fracture strain increased for Ti-210, decreased for Ti-5, and changed negligibly for Ti-30. Comparing high (10^0^ s^−1^) and low (10^−6^ s^−1^) strain rates, twinning occurred more frequently in Ti-30 and Ti-210 at high strain rates, but the frequency did not change in Ti-5. The frequency of 1st order pyramidal slip tended to be higher in Ti-30 and Ti-5 at low strain rates. The higher ductility exhibited by Ti-210 at high strain rates was attributed to the high frequency of twinning. In contrast, the higher ductility of Ti-5 at low strain rates was attributed to the activity of the 1st order pyramidal slip.

## 1. Introduction

Commercially pure titanium (CP-Ti) is widely used in transportation equipment, chemical plants, and other applications owing to its low density, high specific strength, and high corrosion resistance. A fundamental understanding of the strength and ductility of the material is required to realize its safe and long-term use. CP-Ti has a hexagonal close-packed structure at room temperature, and slip system activities occur at the basal, prism, and pyramidal planes. Among these, prism slip is mainly responsible for the plastic strain in general room-temperature deformation. However, the slip direction in prism plane slip is only along the *a*-axis direction and cannot produce strain in the *c*-axis direction. Therefore, twin deformation occurs in the plastic deformation of CP-Ti to carry strain along the *c*-axis direction. The frequency and types of twinning deformation strongly influence not only the strength of the material but also its overall mechanical properties, such as ductility and work-hardening behavior [1,2,3].

Numerous studies on the twinning deformation of CP-Ti have revealed that the frequency and type of active twinning depend on the test temperature, deformation mode (tensile or compressive), and loading direction on the specimen. For example, the frequency of twinning activity increased with deformation at cryogenic temperatures owing to the difference in the temperature dependence of the respective critical resolved shear stress (CRSS) in dislocation slip and twinning deformation [4,5,6,7]. In addition, the deformation temperature affects the type of twinning. At low (200 °C or below) and medium (300–400 °C) temperatures, the $\left\{11\bar{2}2\right\}$ and $\left\{10\bar{1}2\right\}$ twins were active, whereas $\left\{10\bar{1}1\right\}$ twins were active at high (500 °C or above) temperatures [8,9,10,11]. Furthermore, the occurrence of twinning is anisotropic in the microstructure of well-heat-treated pure titanium rolled sheets. In other words, a pure titanium rolled sheet has a transverse direction (TD)-split-type microstructure with [0001] inclined at approximately ±35° from the normal direction (ND) to the TD. In such cases, a tensile test wherein the rolling direction (RD) and the tensile direction are parallel shows that $\left\{11\bar{2}2\right\}$ twins are activated as the main twinning system. In contrast, $\left\{10\bar{1}2\right\}$ twins are active when the TD and the tensile direction are parallel [7,12]. Thus, the twinning deformation behavior depends on the conditions of plastic deformation mode and microstructure. In addition to these factors, the grain size and impurity concentration also affect twinning deformation. For example, the frequency of twinning decreased with decreasing grain size [13]. Moreover, in titanium, the frequency of twinning decreased with increasing oxygen content [14,15].

Investigating the strain-rate dependence of twinning deformation at room temperature is important for understanding the cold workability and the mechanical properties of CP-Ti under conditions of practical usage. For example, Chichili et al. [16] investigated the effect of the strain rate on the mechanical properties and twinning deformation behavior of CP-Ti by performing compression tests at strain rates ranging from 10^−5^ to 10^4^ s^−1^. They found that the work-hardening rate increased with the strain rate and attributed the increase in work-hardening rate to the increased density of twins at higher strain rates. Ahn et al. [17] discussed the relationship between work-hardening and twinning in ASTM Standard Grade 2 CP-Ti using the results of scanning electron microscopy–electron back-scattered diffraction (SEM-EBSD) analysis and obtained results similar to those of Chichili et al. Wang et al. [13] performed high-speed compression tests at 1.5–3.5 × 10^3^ s^−1^ on CP-Ti with an average grain size of 50 µm. The strain-rate dependence of the $\left\{11\bar{2}2\right\}$ twin was larger than that of other twin systems, and the frequency of its occurrence increased with increasing strain rates. Owing to the experimental advantage of being able to easily set a wide range of strain rates, compression tests had used in most studies on the strain-rate dependence of twinning deformation in CP-Ti [16,17,18]. However, the ductility of materials should be evaluated using tensile deformation. Some researchers have reported strain-rate dependence in tensile tests. For example, Yamamoto et al. [19] used CP-Ti with an ultrafine grain size and found that twinning only occurred in a fraction of the specimens handled. Ahn et al. [17] reported the strain-rate dependence of tensile deformation in CP-Ti of common grain sizes; however, in contrast to other studies [20,21], they stated that no twinning was observed in tensile deformation.

In this study, tensile tests were conducted on JIS Class 1 (equivalent to ASTM Grade 1) CP-Ti with three levels of grain size: the strain rate was varied from 10^−6^ to 10^0^ s^−1^, and the relationship between the mechanical properties and the twinning deformation of CP-Ti was investigated. Recently, it has been shown that pyramidal slip activity increases with decreasing strain rates in the creep deformation of the Ti-6Al-4V alloy at room temperature, and the strain-rate dependence of pyramidal slip activity has been experimentally demonstrated [22]. Therefore, in this study, we focused on the strain-rate dependence of both twinning deformation and pyramidal slip.

## 2. Materials and Methods

In this study, two types of CP-Ti sheets with slightly different impurity compositions were used. Both the sheets were JIS Standard Class 1 (equivalent to ASTM Standard Grade 1), and they were both treated as pure titanium sheets without distinction. The chemical compositions of the two types of CP-Ti sheets are listed in Table 1. To adjust the grain size, the sheets were cold-rolled and then annealed in a vacuum at 800, 650, and 500 °C for 4 h, followed by furnace cooling. The average grain sizes of the samples labelled Ti-210, Ti-30, and Ti-5 were 210, 30, and 5 µm, respectively.

**Figure 1:**
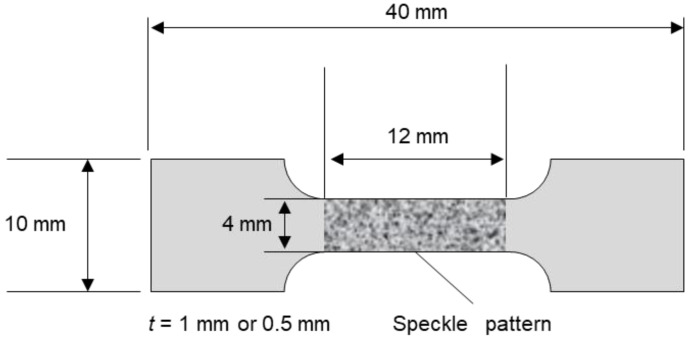
Schematic of the specimen used in the tensile test.

The CP-Ti sheets were cut into tensile specimens using an electrical discharge machine; a schematic of the specimen is shown in Figure 1. The specimens were prepared such that the ND of the plate surface was parallel to the ND and the tensile direction was parallel to the RD. The specimen thickness of both Ti-30 and Ti-5 was approximately 0.5 mm. Zhu et al. [23] studied the effects of grain size and specimen thickness on the frequency of twinning. They reported that specimens containing three or fewer grains through the thickness had a lower probability of twinning in comparison with bulk specimens. To investigate the twinning behavior of CP-Ti sheets as a bulk material, the specimen thickness of Ti-210 was 1 mm such that there were at least four grains in the specimen thickness direction.

The specimens were subjected to tensile testing at room temperature at three different strain rates ($\dot{\varepsilon }$ = 10^−6^, 10^−4^, and 10^−2^ s^−1^) using an Instron-type tensile testing machine (Autograph, Shimadzu Corp., Kyoto, Japan.); a hydraulic tensile testing machine (Servopulser, Shimadzu Corp.) was used to conduct tensile tests at a strain rate of $\dot{\varepsilon }$ = 10^0^ s^−1^. For tensile tests at strain rates $\dot{\varepsilon }$ = 10^−6^, 10^−4^, and 10^−2^ s^−1^, the digital image correlation (DIC) method was used to measure the strain. In the Instron-type tensile testing machine used in this study, the slope of linear relationship between stress and strain at the elastic deformation region was small due to the influence of the testing machine rigidity, specimen fixation, and fixture deformation. Therefore, the DIC method was employed to evaluate the strain of the specimen only. For the DIC method, a speckle pattern was drawn using black and white lacquer sprays. During deformation, the changes in the pattern were captured at intervals of 0.1–10 s by optical microscopy. However, at a strain rate of $\dot{\varepsilon }$ = 10^0^ s^−1^, it was not possible to obtain clear images of the pattern using an optical microscope. Therefore, the strain was estimated using the displacement obtained from the testing machine. Tensile interruption tests were performed at strain rates $\dot{\varepsilon }$ = 10^−6^ and 10^0^ s^−1^. In these interruption tests, the plastic strain was calculated directly by measuring the change, before and after deformation, in the distance between two indentations by a Vickers hardness tester (~10 mm before deformation). Each tensile test was performed twice to confirm the reproducibility of strength and ductility.

Secondary electron (SE) images were obtained by SEM (ULTRA 55, Carl-Zeiss AG, Oberkochen, Germany; Scios, Thermo-Fisher-Scientific Inc., Waltham, MA, USA) to observe the microstructure. EBSD was used to analyze the crystal orientation. EBSD data were acquired using an OIM system (Ametek Inc, Berwyn, PA, USA). The acceleration voltage was 15 kV, and the step size was varied from 0.1 to 1 µm depending on the grain size. The specimens used for observation were wet-polished with emery paper (from #80 to #3000). This was followed by electropolishing in an electrolytic solution of methanol:perchloric acid = 95:5 (volume ratio) under the conditions of a voltage of 10 V, temperature of −40 °C, and current of 0.5 A.

## 3. Results

### 3.1. Initial Microstructure

**Figure 2:**
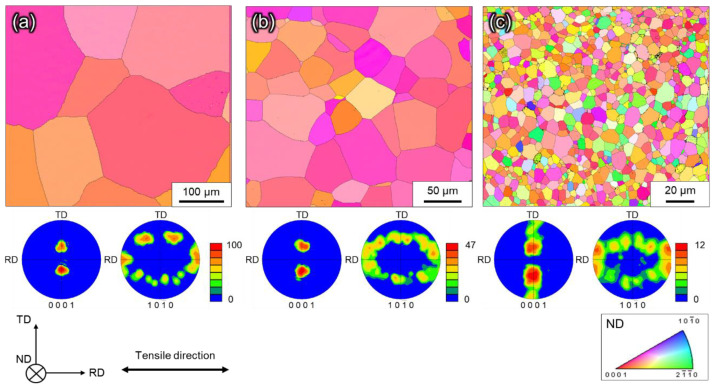
Crystal orientation distribution maps of initial microstructure (ND), (0001) pole figure, and $\left\{10\bar{1}0\right\}$ pole figure of (**a**) Ti-210, (**b**) Ti-30, and (**c**) Ti-5.

Figure 2 shows the crystal orientation distribution map, (0001) pole figure, and $\left\{10\bar{1}0\right\}$ pole figure of the microstructure before tensile testing. The left and right directions on the paper correspond to the tensile and RD, respectively. Each crystal orientation distribution map shows the crystal orientation relative to the direction of observation (=ND) based on the color inside the standard stereographic triangle. The crystal orientation distribution map indicated that no twinning was observed in the initial microstructure. As can be seen from the pole figures, the initial texture exhibited characteristics typical of a well-annealed cold-rolled plate. In other words, (0001) is inclined at ±35° from the ND to the TD, and the $\left\{10\bar{1}0\right\}$ pole figure is distributed along a great circle centered at the (0001) pole. However, its intensity is slightly lower in Ti-5.

### 3.2. Evaluation of Mechanical Properties by Tensile Testing

**Figure 3:**
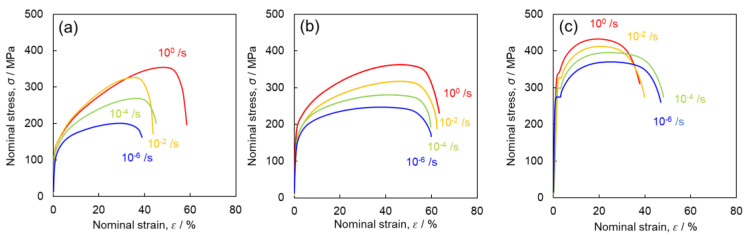
Nominal stress-nominal strain curves of (**a**) Ti-210, (**b**) Ti-30, and (**c**) Ti-5 obtained from tensile tests performed at room temperature at strain rates ranging from 10^−6^ to 10^0^ s^−1^.

Figure 3 shows an example of the nominal stress-nominal strain curves for the tensile tests at strain rates ranging from 10^−6^ to 10^0^ s^−1^. The 0.2% proof stress $\sigma_{0.2}$, ultimate tensile strength $\sigma_{\mathrm{UTS}}$, and fracture strain $\varepsilon_{f}$ are summarized in Table 2. It is evident from Figure 3 and Table 2 that $\sigma_{0.2}$ and $\sigma_{\mathrm{UTS}}$ increase with increasing strain rates in all samples. At the same strain rate, the values of $\sigma_{0.2}$ and $\sigma_{\mathrm{UTS}}$ are higher for finer grain sizes. With an increase in strain rate, $\varepsilon_{f}$ increases for Ti-210 and decreases for Ti-5, and the variation is negligible for Ti-30.

The strain-rate sensitivity m of each sample to $\sigma_{0.2}$ and $\sigma_{\mathrm{UTS}}$ was estimated using the following equation:

(1) $$m=\frac{d\ln\sigma }{d\ln\dot{\varepsilon }}\;,$$

The m values calculated for each sample are listed in Table 3. For Ti-210, the m value of $\sigma_{\mathrm{UTS}}$ is larger than the m value of $\sigma_{0.2}$. Therefore, it can be inferred that the strain rates play a significant role in the work-hardening behavior of this sample. In contrast, for Ti-5, the m value of $\sigma_{0.2}$ is larger than the m value of $\sigma_{\mathrm{UTS}}$. Hence, in this sample, the strain rates are more strongly related to the yield behavior than to the work-hardening behavior.

For Ti-30, the m values of $\sigma_{0.2}$ and $\sigma_{\mathrm{UTS}}$ are comparable, indicating that the strain rate affects both yield and work-hardening behaviors. The m values of $\sigma_{\mathrm{UTS}}$ for Ti-210 and Ti-30 are larger than that of Ti-5. These results suggest that different mechanisms are involved in the effects of strain rates on the mechanical properties of Ti-210 and Ti-5, and that both mechanisms are involved in Ti-30, whose grain size lies between those of Ti-210 and Ti-5.

### 3.3. Evaluation of Mechanical Properties by Tensile Testing

**Figure 4:**
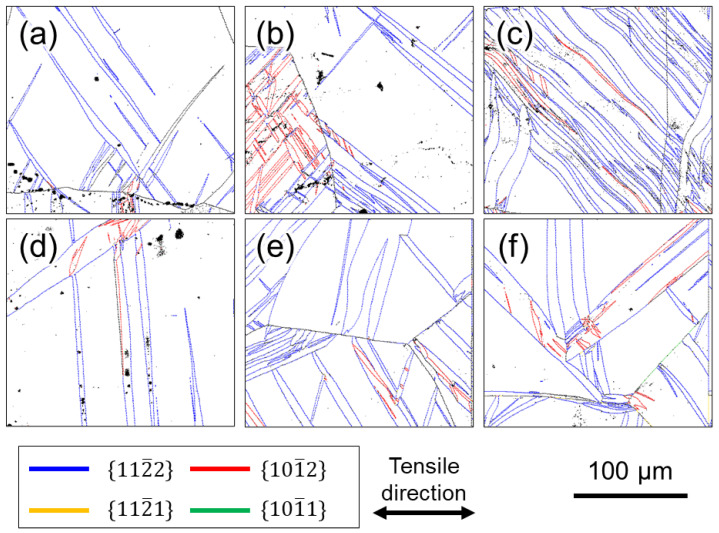
GB maps of Ti-210 after deformation. Interrupted specimens at (**a**) 12%, (**b**) 14%, and (**c**) 22% strain in high strain rates, and (**d**) 10%, (**e**) 14%, and (**f**) 18% strain in low strain rates.

**Figure 5:**
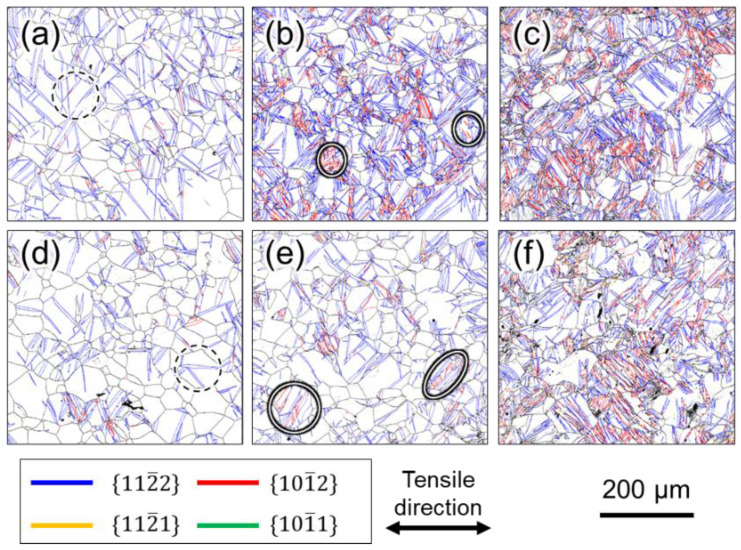
GB maps of Ti-30 after deformation. Interrupted specimens at (**a**) 6%, (**b**) 16%, and (**c**) 30% strain in high strain rates, and (**d**) 8%, (**e**) 14%, and (**f**) 30% strain in low strain rates. The areas circled in white in (**b**,**e**) were confirmed by $\left\{10\bar{1}2\right\}\langle 10\bar{1}\bar{1}\rangle$ twins.

**Figure 6:**
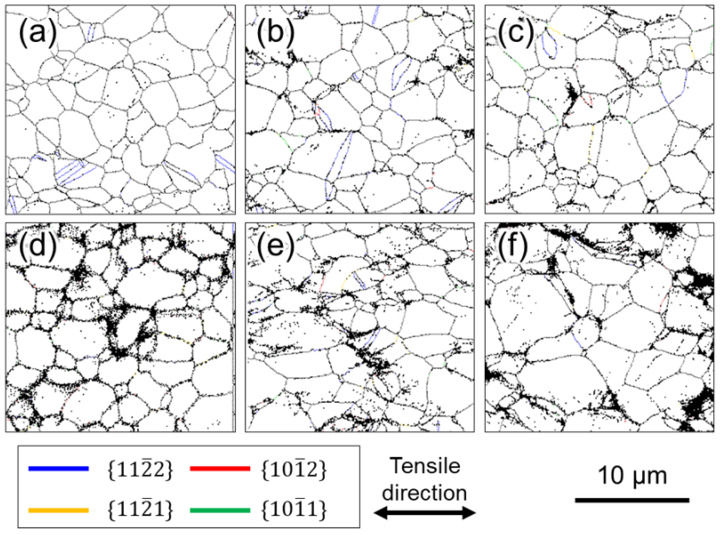
GB maps of Ti-5 after deformation. Interrupted specimens at (**a**) 7%, (**b**) 14%, and (**c**) 24% strain in high strain rates, and (**d**) 8%, (**e**) 14%, and (**f**) 24% strain in low strain rates.

Figure 4, Figure 5 and Figure 6 show the grain boundary maps (GB maps) after tensile testing at 10^0^ s^−1^ and 10^−6^ s^−1^. Figure 4 shows Ti-210 specimens interrupted at (a) 12%, (b) 14%, and (c) 22% strain at high strain rates, and (d) 10%, (e) 14%, and (f) 18% strain at low strain rates. Figure 5 shows Ti-30 specimens interrupted at (a) 6%, (b) 16%, and (c) 30% strain at high strain rates, and (d) 8%, (e) 14%, and (f) 30% strain at low strain rates. Figure 6 shows Ti-5 specimens interrupted at (a) 7%, (b) 14%, and (c) 24% strain at high strain rates, and (d) 8%, (e) 14%, and (f) 24% strain at low strain rates. In all the figures, the left and right directions on the paper correspond to the tensile direction during tensile testing. In the GB maps, the random high-angle (defined as 15° or greater) grain boundaries are shown in black, and the grain boundaries classified as the $\left\{10\bar{1}2\right\}\langle 10\bar{1}\bar{1}\rangle$, $\left\{11\bar{2}2\right\}\langle 11\bar{2}\bar{3}\rangle$, $\left\{10\bar{1}1\right\}\langle 10\bar{1}\bar{2}\rangle$, and $\left\{11\bar{2}1\right\}\langle 10\bar{2}\bar{6}\rangle$ twin boundaries are shown in red, blue, green, and yellow, respectively. For the twin boundaries, the tolerance angle from the ideal value of the *K*_1_ plane and orientation difference was set to between 10° and 15°. Normally, the ideal tolerance angle for a twin boundary is 5°. However, the tolerance angle of 5° misses the twin boundary because the actual crystal orientation relationship between parent and twin phases changes due to deformation. Therefore, the tolerance angle in this study was set to a higher value. Figure 4 and Figure 5 show that twinning occurred in the grains of Ti-210 and Ti-30 at both high (10^0^ s^−1^) and low strain rates (10^−6^ s^−1^), and the frequency of twinning increased as the deformation progressed. The twins are lens-shaped: thicker at the center and thinner toward the tip. At both deformation rates, $\left\{11\bar{2}2\right\}\langle 11\bar{2}\bar{3}\rangle$ twins preferentially formed at low strains. As the deformation progressed, $\left\{10\bar{1}2\right\}\langle 10\bar{1}\bar{1}\rangle$ twins formed more frequently. The $\left\{11\bar{2}2\right\}\langle 11\bar{2}\bar{3}\rangle$ twins occurred homogenously in grains of any orientation. In contrast, $\left\{10\bar{1}2\right\}\langle 10\bar{1}\bar{1}\rangle$ twins were generally a result of secondary twinning inside the $\left\{11\bar{2}2\right\}\langle 11\bar{2}\bar{3}\rangle$ twins, as observed in the grains circled by a solid white line in Figure 5b,e. As shown in Figure 2, this specimen has a TD-split texture with [0001] tilted by approximately ±35° from the ND to the TD. When $\left\{11\bar{2}2\right\}\langle 11\bar{2}\bar{3}\rangle$ twinning—wherein the *c* axis rotates by approximately 64°—occurs in some grains having such a texture, the *c* axis inside the twins becomes almost parallel to the tensile direction. Consequently, inside such $\left\{11\bar{2}2\right\}\langle 11\bar{2}\bar{3}\rangle$ twins, $\left\{10\bar{1}2\right\}\langle 10\bar{1}\bar{1}\rangle$ twins with tensile twinning properties are likely to form as secondary twins during subsequent deformation. The order of occurrence of such twinning deformation has been reported for CP-Ti under tensile deformation [7,21] and is opposite to that reported for compressive deformation [12,24]. In this study, the occurrence of $\left\{11\bar{2}1\right\}\langle 10\bar{2}\bar{6}\rangle$ twins was minimal under all deformation conditions for every sample, and no $\left\{10\bar{1}1\right\}\langle 10\bar{1}\bar{2}\rangle$ twins were observed. The $\left\{11\bar{2}2\right\}\langle 11\bar{2}\bar{3}\rangle$ and $\left\{10\bar{1}2\right\}\langle 10\bar{1}\bar{1}\rangle$ twins mostly occurred at the grain boundaries, and twins of the same type formed in multiple parallel layers within a grain. Tsukamoto et al. [8] reported that these twins occur at grain boundaries during room-temperature deformation. The introduction of these twins indicates that the grain size gradually becomes finer with increasing deformation. Notably, at similar strains, both $\left\{11\bar{2}2\right\}\langle 11\bar{2}\bar{3}\rangle$ and $\left\{10\bar{1}2\right\}\langle 10\bar{1}\bar{1}\rangle$ twins occurred more frequently at high strain rates (10^0^ s^−1^) than at low strain rates (10^−6^ s^−1^). Therefore, at high strain rates (10^0^ s^−1^), the grain refinement associated with twinning is significantly enhanced.

**Figure 7:**
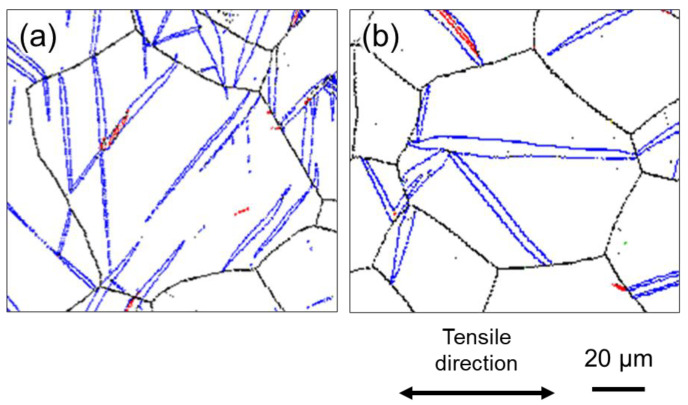
Enlarged GB maps for the areas enclosed by the black dashed circle in Figure 5a,d corresponding to (**a**) high strain rate (10^0^ s^−1^) and (**b**) low strain rate (10^−6^ s^−1^), respectively.

In addition, it is important to focus on the shape of the twins. Figure 7a,b show expanded views of the GB map in the grains circled by the black dashed lines in Figure 5a,d, respectively. At high strain rates (10^0^ s^−1^) (Figure 7a), the aspect ratio of the twins is large, whereas at low strain rates (10^−6^ s^−1^) (Figure 7b), the twins are rather thick in the width direction. The average value of the aspect ratio for the twins observed in the entire field of view of Figure 5a,d were 8.9 and 5.9, respectively, indicating that thin twins are more likely to be generated under deformation at high strain rates. Thus, it can be concluded that a large number of thin twins tend to be generated at high strain rates (10^0^ s^−1^) and that the thin twins more effectively subdivided the crystal grains during deformation.

Next, we focus on the microstructural observations of Ti-5 shown in Figure 6. In contrast to Ti-210 and Ti-30, no difference in the frequency of twinning in Ti-5 was observed under different test conditions. As in Ti-210 and Ti-30, the twinning systems observed in Ti-5 were $\left\{11\bar{2}2\right\}\langle 11\bar{2}\bar{3}\rangle$ twins and $\left\{10\bar{1}2\right\}\langle 10\bar{1}\bar{1}\rangle$ twins, and almost no other twins were observed.

**Figure 8:**
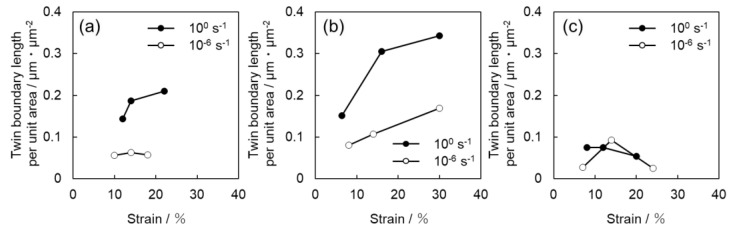
Strain dependence of the twin boundary length per unit area for (**a**) Ti-210, (**b**) Ti-30, and (**c**) Ti-5.

**Figure 9:**
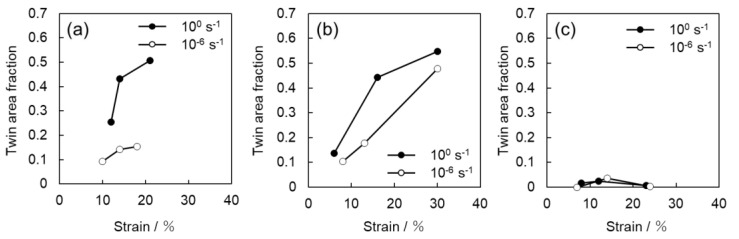
Strain dependence of the twin area fraction for (**a**) Ti-210, (**b**) Ti-30, and (**c**) Ti-5.

Figure 8 summarizes the relationship between the frequency of twinning and strain for all samples, which are shown in Figure 4, Figure 5 and Figure 6. In Figure 8, the twin boundary length per unit area was used as an indicator of the frequency of twinning. This value is directly related to the progress of grain refinement owing to the introduction of twins. In Figure 8, the boundary lengths of $\left\{11\bar{2}2\right\}\langle 11\bar{2}\bar{3}\rangle$ twins and $\left\{10\bar{1}2\right\}\langle 10\bar{1}\bar{1}\rangle$ twins are summed. Figure 9 shows the relationship between the area fraction of twin and strain. The area fraction of twin is often used as a parameter to evaluate the frequency of twinning. In this study, the area fraction of twin was calculated as the percentage of the area inside the twinning boundaries of $\left\{11\bar{2}2\right\}\langle 11\bar{2}\bar{3}\rangle$ twins and $\left\{10\bar{1}2\right\}\langle 10\bar{1}\bar{1}\rangle$ twins on the OIM system. For Ti-210 and Ti-30, the twin boundary length tends to increase with increasing strain for both high (10^0^ s^−1^) and low (10^−6^ s^−1^) strain rates, as is qualitatively demonstrated in Figure 4 and Figure 5. In other words, the number density of twins increases. The twin boundary length unit per area is clearly higher at high strain rates (10^0^ s^−1^) than at low strain rates (10^−6^ s^−1^), as shown Figure 8.

In Figure 9, the twinning area fraction also shows the same tendency as that observed in Figure 8. While the twin boundary length per unit area of Ti-30 is higher than that of Ti-210, the twin area fraction of Ti-210 is slightly higher than that of Ti-30. These results suggest that the number density of twins in Ti-210 is smaller than that in Ti-30, but each twin is thicker. On the other hand, in Ti-5, the frequency of twinning is low at both high (10^0^ s^−1^) and low (10^−6^ s^−1^) strain rates.

## 4. Discussion

### 4.1. Effect of Strain Rates on Twinning

**Figure 10:**
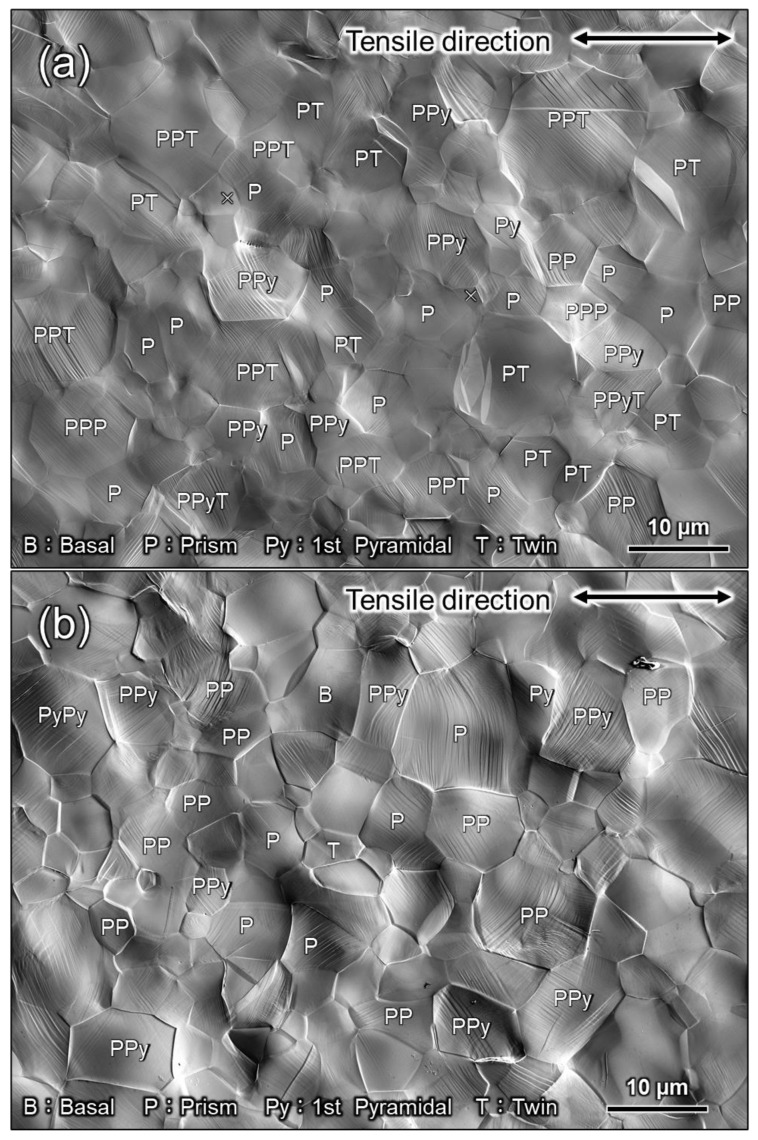
SEM images of Ti-30 after deformation by approximately 15% plastic strain at (**a**) high (10^0^ s^−1^) and (**b**) low (10^−6^ s^−1^) strain rates. *B*, *P*, *Py*, and *T* indicate that the basal slip, prism slip, 1st order pyramidal slip, and twinning were active, respectively.

**Figure 11:**
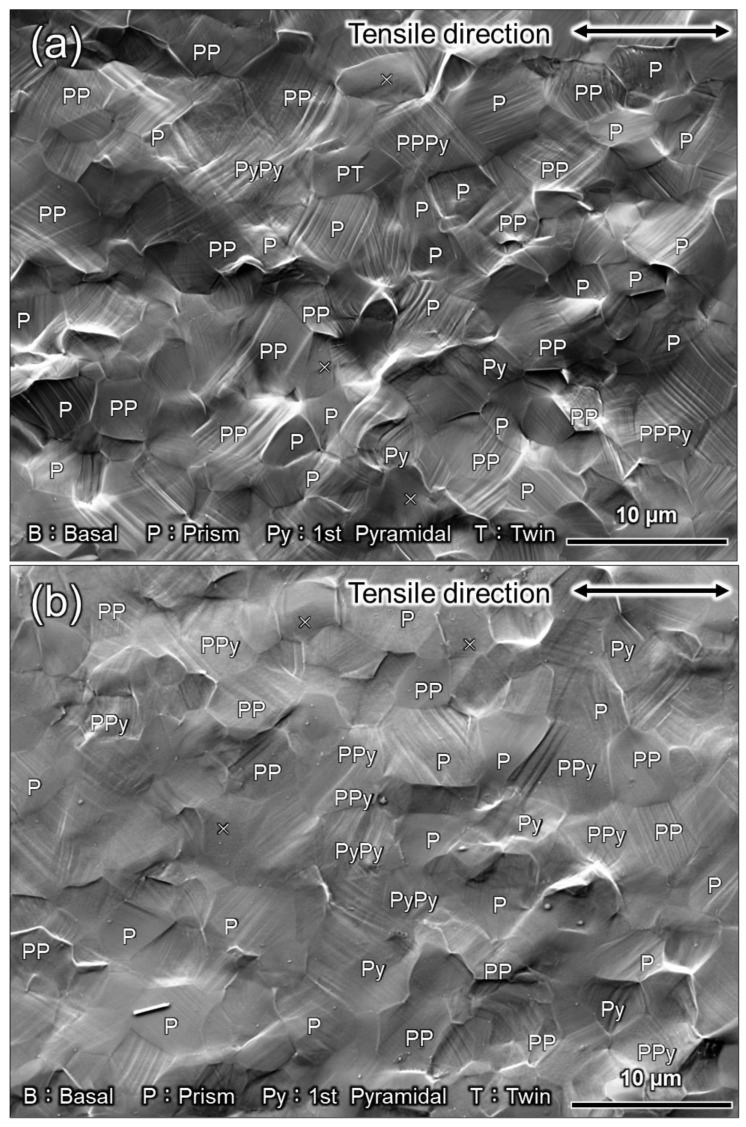
SEM images of Ti-5 after deformation by (**a**) approximately 18% plastic strain at high strain rates (10^0^ s^−1^) and (**b**) approximately 14% plastic strain at low strain rates (10^−6^ s^−1^). *B*, *P*, *Py*, and *T* indicate that the basal slip, prism slip, 1st order pyramidal slip, and twinning, respectively.

**Figure 12:**
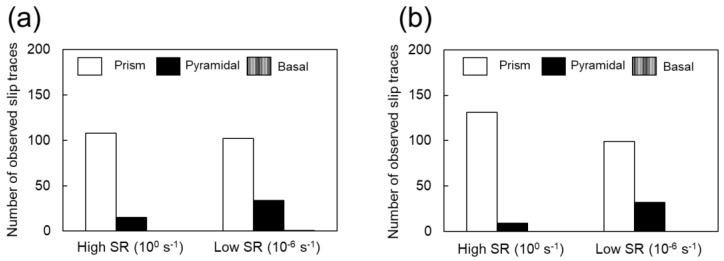
Number of observed slip traces based on Figure 10 and Figure 11 in (**a**) Ti-30 and (**b**) Ti-5 deformed at high (10^0^ s^−1^) and low (10^−6^ s^−1^) strain rates.

**Figure 13:**
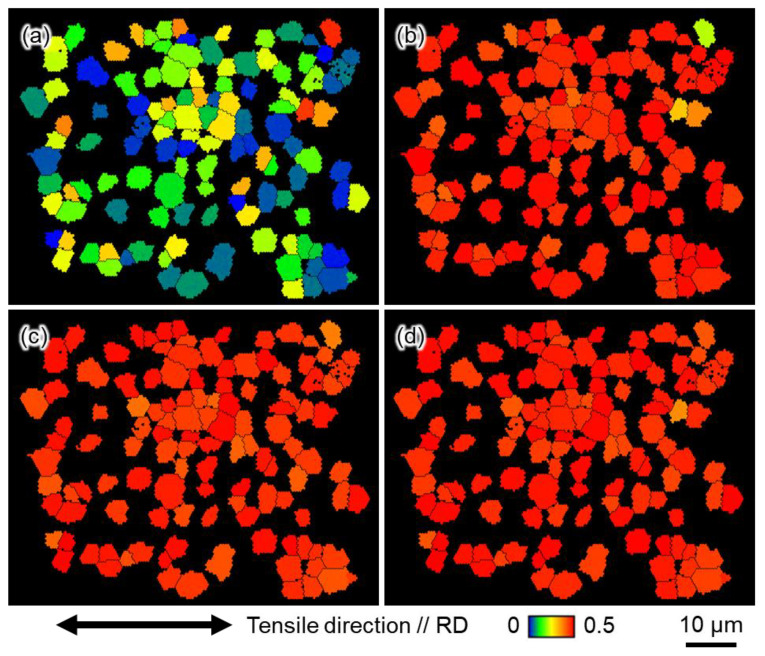
SF maps in the tensile direction in Ti-5 of (**a**) basal, (**b**) prism, (**c**) 1st order pyramidal 〈a〉, and (**d**) 1st order pyramidal 〈a+c〉 dislocation slips.

In CP-Ti, prismatic 〈a〉 dislocation slip is the principal slip system at room temperature. However, this alone is not sufficient for plastic deformation to proceed to an arbitrary shape: either the slip system that includes the *c*-component, or the twinning, or both must be active concurrently with the principal slip. In other words, to discuss the occurrence of twinning, an understanding of the dislocation motion that is concurrently active is necessary. For example, the increased frequency of twinning at cryogenic temperatures is attributed to the temperature dependence of the CRSS of the dislocation slip [25,26]. In a study that used crystal plasticity finite element analysis to investigate the effect of strain rates on the CRSS of dislocation slip, Rodríguez-Galan et al. [27] showed that CRSS increases with higher strain rates in nanostructured pure titanium. However, there are few reports on the dependence of CRSS on strain rate in pure titanium with normal grain size. Table 3 shows the strain rate sensitivities of $\sigma_{0.2}$ and $\sigma_{\mathrm{UTS}}$. Dislocation motion and twinning are synergistically involved in $\sigma_{\mathrm{UTS}}$. In contrast, $\sigma_{0.2}$ should be strongly related to the CRSS of the main slip, i.e., the prism 〈a〉 dislocation slip. In other words, the strain-rate dependence of $\sigma_{0.2}$ indicates that the CRSS of the prism 〈a〉 dislocation slip is strain-rate-dependent. Hence, the increased CRSS of this dislocation slip can be considered as a factor that contributes to the increased frequency of twinning at higher strain rates. Next, we consider the contribution of dislocation motion other than that of the prism slip. As shown in Figure 8 and Figure 9, the frequency of twinning increases with increasing strain. In titanium, as deformation progresses, dislocation activities other than prism slip may occur, and these activities may affect the frequency of twinning. Figure 10 shows SEM-SE images of Ti-30 deformed up to approximately 15% at high (10^0^ s^−1^) and low (10^−6^ s^−1^) strain rates. Figure 11 shows the SEM-SE images of Ti-5 deformed up to approximately 18% at high strain rates (10^0^ s^−1^) and 14% at low strain rates (10^−6^ s^−1^). In both Figure 10 and Figure 11, slip lines that correspond to active dislocation slips in each grain can be clearly observed. By focusing on the slip lines, it is possible to distinguish grains with a single slip from grains with multiple slips. The following procedure was used for slip line trace analysis [28]: EBSD measurements were performed in the same field of view as the SE image in Figure 10 and Figure 11. For the grains with clearly visible slip lines in the SE image, each slip plane was drawn using the slip plane notation function in the OIM system. The slip plane is represented by a single or multiple intersecting lines, with one line for the basal plane, three lines for the prismatic plane, and six lines for the pyramidal plane. Comparing slip lines on the SE image and slip plane traces drawn by the OIM system, the one that is parallel is the slip plane of the active slip system. Slip line tracing analysis was performed on 80 and 100 grains for each condition for Ti-30 and Ti-5, respectively. The corresponding symbols are shown in the figure, where *P* indicates the prism slip, *B* indicates the basal slip, and *Py* indicates the 1st order pyramidal slip. Multiple symbols indicate the occurrence of multiple slips. *T* indicates twinning and cross marks indicate that the slip line could not be confirmed on SE images. The 1st order pyramidal slip is active in both 〈a〉 dislocation slip and 〈a+c〉 dislocation slip but slip line tracing analysis cannot distinguish between the two. A comparison of the number of slip systems observed in Figure 10 and Figure 11 is shown in Figure 12. In the case where multiple traces of prism slip are observed in a grain, e.g., *PP* in Figure 10, the number of *P* is counted as two. At both high (10^0^ s^−1^) and low (10^−6^ s^−1^) strain rates, prism slip was the dominant dislocation slip. The activity of the basal slips was negligible. Figure 13 shows the Schmid factor (SF) maps of (a) basal, (b) prism, (c) pyramidal 〈a〉, and (d) pyramidal 〈a+c〉 slips for Ti-5. The observation area of Figure 13 matches that of Figure 11b, and the grains extracted are the those with traced slip planes in Figure 11b. The SF maps show that the SFs are high for prism, pyramidal 〈a〉, and pyramidal 〈a+c〉 slips in most grains, but low for basal slips. This tendency is well explained by the fact that little basal slip activity is observed in Figure 12.

We now focus on the behavior of pyramidal slip. Pyramidal slip has a higher CRSS than prism slip [29], which limits its activity. However, it is the only active dislocation slip that contain a *c*-component displacement. It has also been reported that the frequency of the pyramidal slip gradually increases as the deformation progresses [19]. Figure 10 and Figure 11 show that pyramidal slip activity was observed in this study. Notably, the frequency of pyramidal slip activity increased at low strain rates (10^−6^ s^−1^). This is probably related to the thermal activation process of the pyramidal slip, which is similar to the behavior reported for Ti-6Al-4V alloys [22]. The activation of pyramidal slip with a *c*-component is expected to contribute to satisfying the strain compatibility and relieving the deformation constraint near grain boundaries and the associated stress concentration. The $\left\{11\bar{2}2\right\}\langle 11\bar{2}\bar{3}\rangle$ and $\left\{10\bar{1}2\right\}\langle 10\bar{1}\bar{1}\rangle$ twins observed in this study were both triggered by stress and/or strain concentrations near the grain boundary [8]. In this study, twinning activity was observed in Ti-210 and Ti-30. The decrease in the frequency of twinning with decreasing strain rates can be attributed to the fact that the pyramidal slip weakened the stress and/or strain concentrations. On the other hand, in Ti-5, the frequency of twinning is almost independent of strain rates (Figure 8 and Figure 9). In general, it is reported that the twinning deformation of titanium has a stronger grain size dependence than that of Mg and other materials [30]. Moreover, the occurrence of twinning is suppressed in fine grain sizes, and a coarser grain size corresponds to a higher frequency of twinning [31]. This is because the stress concentration at grain boundaries is enhanced in coarse grains. Therefore, the clear suppression of twinning in Ti-5 is attributed to grain refinement. Specifically, pyramidal slips are activated with decreasing strain rates, as shown in Figure 12. The activation of these pyramidal slips has a significant effect on the ductility of Ti-5, with lower strain rates resulting in higher ductility.

### 4.2. Effect of Grain Size on Twinning and Work-Hardening Behavior

**Figure 14:**
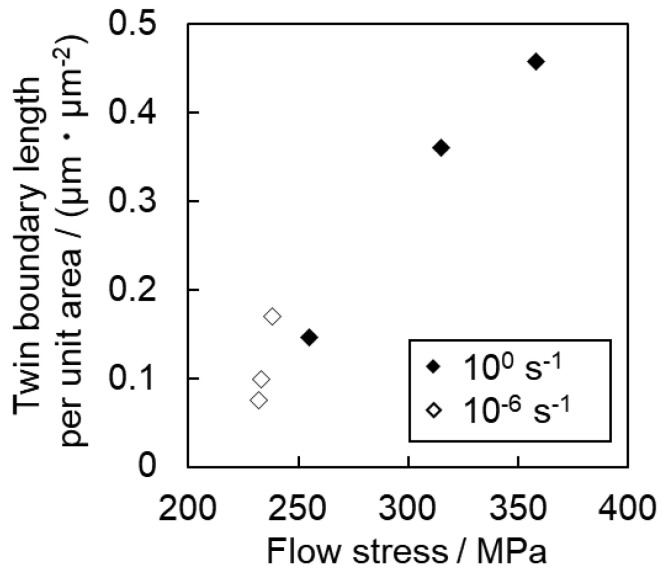
Relationship between twin boundary length per unit area and flow stress in Ti-30.

**Figure 15:**
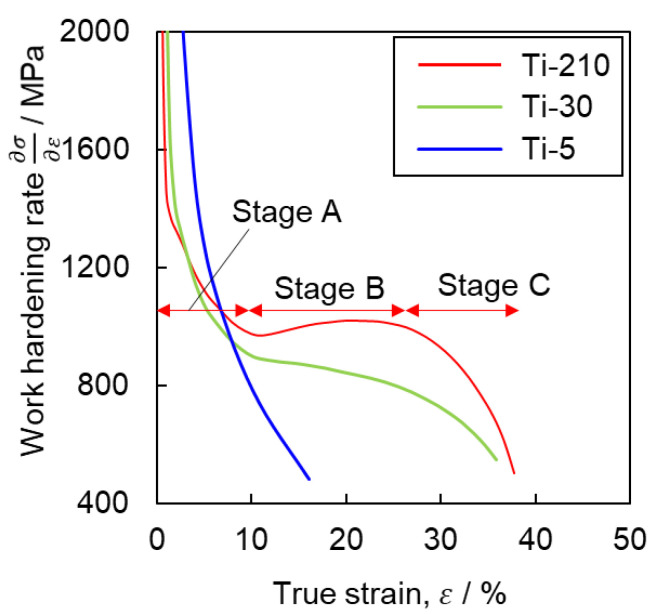
Work-hardening rate–true strain curves obtained from tensile tests at high strain rates (10^0^ s^−1^).

Figure 14 shows the relationship between the length of the twin boundary per unit area and the flow stress at the interruption of tensile testing in Ti-30. There is a clear positive correlation between twin boundary length and flow stress. Tsukamoto et al. [32] reported that grain refinement by the introduction of twins is effective in increasing dislocation density, which leads to excellent work-hardening. Accordingly, Figure 15 shows a comparison of the work-hardening rate of each sample during deformation at high strain rates (10^0^ s^−1^), which was the strain rate that showed the best work-hardening result in this study. Ti-5 exhibits the general behavior of a decreasing work-hardening rate with increasing strain. In contrast, for Ti-210 and Ti-30, the variation in work-hardening rate followed a specific S-shaped curve. Similar trends were reported by Salem et al. [2,33] and Ahn et al. [17]. Similar to the results of Salem et al. [2,33], the S-shaped curve herein is classified into stages A, B, and C, as shown in Figure 15. Stage A corresponds to the region where the work-hardening rate decreases owing to the dynamic recovery of the dislocations. Stage B corresponds to the introduction of twins. This is evident from the fact that Stage B does not exist in Ti-5, wherein the frequency of twinning is lower. In Stage C, crystal refinement progresses owing to the occurrence of deformation twinning, and the occurrence of twins saturates. Thus, the dynamic recovery of dislocations alone contributes to the work-hardening rate. Ti-210 shows a higher work-hardening rate at the same strain compared with Ti-30, reaching a maximum value during Stage B. Additionally, from Figure 3, discontinuous yielding is observed in Ti-5. This is often observed in titanium with an average grain size of several hundred nm to several µm [34,35,36]. Such discontinuous yielding has the effect of increasing the work hardening rate. However, in the present study, the work hardening rate of Ti-5 decreases monotonically. This may be attributed to small number of measurement points under our experimental conditions. At high strain rates (10^0^ s^−1^), the time to rupture was approximately 0.3 s, and the measurement interval was 0.01 s. Therefore, the s-s curve of 10^0^ s^−1^ in Figure 3c also shows less clear discontinuous yielding behavior compared to the other conditions.

**Figure 16:**
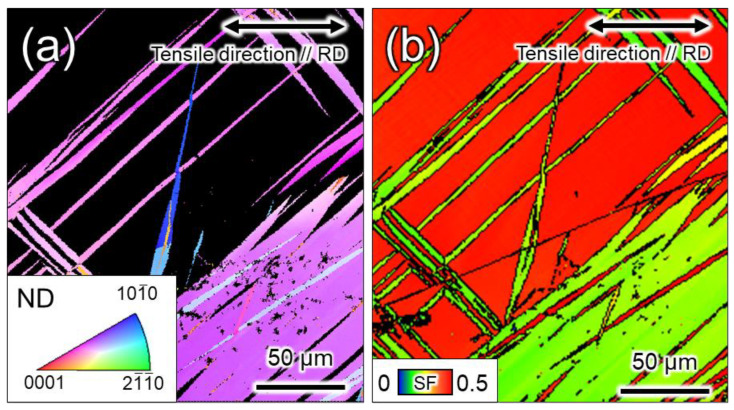
(**a**) Crystal orientation distribution map highlighting only the inside of the twin boundary and (**b**) SF map of prism dislocation slip for the tensile direction in Ti-210 deformed 14% at high strain rates (10^0^ s^−1^).

Next, we will discuss the factors that increased the work hardening rate in Ti-210 and Ti-30 by introduction of twinning. In general, the twinning-induced plasticity effect refers to the introduction of fine deformation twins during plastic deformation, where the twin boundaries become an obstacle to dislocation movement and work hardening increases [37]. The work-hardening rate would be large in Ti-30, where many fine twins are introduced. However, in this study, Ti-210 exhibited the highest work-hardening rate. Tsukamoto et al. [29] reported that the promotion of work hardening by twinning deformation can be explained by not only grain refinement but also the change in the Taylor factor due to the change in the texture. Figure 16 shows the (a) crystal orientation distribution map and (b) SF map of prism 〈a〉 in the tensile direction for Ti-210 deformed by 12% at high strain rates; in (a), only grains within the twin boundary are highlighted. It is apparent that the SF for prism 〈a〉 is lower within the twin boundary than in the parent phase. This suggests that the introduction of twins in titanium has two effects on work hardening: one is that the twin boundary acts as a barrier to dislocation motion, increasing the work-hardening rate. The other is that the crystal orientation inside the twin boundary reduces the main prism 〈a〉 dislocation slip activity, which enhances apparent work hardening. In Ti-210, the twin area fraction is slightly higher than in Ti-30. Therefore, it is considered that the latter effect is largely manifested, resulting in a high work-hardening rate.

The discussion thus far indicates that twinning plays an important role in work-hardening. This also implies that twinning is closely related to ductility in the tensile tests. As shown in Table 2, Ti-210 exhibited higher ductility at higher strain rates (10^0^ s^−1^). This can be attributed to the fact that an increase in the frequency of twinning increases the work-hardening ability and promotes uniform deformation. However, for Ti-5, where the frequency of twinning was lower, the fracture strain increased with a lower strain rate. This is because of the activation of pyramidal slip, as shown in Figure 12. In Ti-5, pyramidal slip activity is more demanding than in other samples because of the high flow stress due to the fine grains and the suppression of twinning. Because pyramidal slip becomes more active at lower strain rates, it is deduced that Ti-5 exhibits a higher fracture strain at lower strain rates. In other words, two phenomena are involved in controlling the ductility of pure titanium: deformation twinning and the activation of pyramidal slip. The dominant factor is determined by the strain rates and grain size. In Ti-30, the fracture strain was constant regardless of the strain rate. This is because of the contribution of twinning at high strain rates and pyramidal slip at low strain rates.

Finally, we discuss the m values for each of the samples in Table 3. The positive value of m for $\sigma_{0.2}$ in Table 3 for all specimens indicates that the prism slip is strain rate dependent. Strictly speaking, however, the $\sigma_{0.2}$ is also affected by the very early work-hardening behavior, i.e., the process of dislocation density increase. In Figure 12, the contribution of pyramidal slip activity to deformation is suggested, indicating a clear strain-rate dependence of pyramidal slip activity. Tsukamoto et al. [8] reported that pyramidal 〈a+c〉 slip activity becomes more active with decreasing grain size. This is one of the reasons why the m values of Ti-30 and Ti-5 are larger than those of Ti-210. However, the mechanism by which the $\sigma_{0.2}$ decreases at lower strain rates, where pyramidal slip would be more active, is unclear and requires further detailed investigation. Next, we note that the m values of $\sigma_{\mathrm{UTS}}$ for Ti-30 and Ti-210 show a large dependence on strain rate. As shown in Figure 4, this is presumably due to the increase in the frequency of twinning with increasing strain rate. In other words, as shown in Figure 15, it can be concluded that the increase in work hardening rate due to twinning is directly responsible for the *m* value of $\sigma_{\mathrm{UTS}}$. This is supported by the fact that the m values of $\sigma_{\mathrm{UTS}}$ does not show a large value in Ti-5, where the twinning rate does not show a strain rate dependence.

## 5. Conclusions

To investigate the relationship between mechanical properties and twinning behavior, tensile tests were performed on CP-Ti (Ti-210, Ti-30, and Ti-5) with different grain sizes (210, 30, and 5 µm, respectively) at strain rates varying from 10^−6^ to 10^0^ s^−1^. The following conclusions were obtained:

- In all the samples, the 0.2% proof stress and ultimate tensile strength increased with an increase in the strain rate. At the same strain rates, the samples with finer grain sizes exhibited higher strengths. The variation in fracture strain was different for each sample: for Ti-210, the fracture strain increased with increasing strain rates. In contrast, for Ti-5, the fracture strain decreased with increasing strain rates. For Ti-30, the variation in the fracture strain with strain rate was negligible.
- In Ti-30 and Ti-210, the frequency of twinning increased with an increase in the strain. At low strains, $\left\{11\bar{2}2\right\}\langle 11\bar{2}\bar{3}\rangle$ twins were preferentially formed, and as deformation progressed, $\left\{10\bar{1}2\right\}\langle 10\bar{1}\bar{1}\rangle$ twins formed more frequently. At high strain rates (10^0^ s^−1^), the frequency of twinning was higher than that at low strain rates (10^−6^ s^−1^), and thin twins tended to form more frequently. For Ti-5, the frequency of twinning did not change with an increase in the strain.
- When Ti-30 was deformed at high (10^0^ s^−1^) and low (10^−6^ s^−1^) strain rates up to approximately 15%, the frequency of pyramidal slips tended to increase at low strain rates (10^−6^ s^−1^). This change in the frequency of the pyramidal slip activity affects the frequency of twinning.
- In Ti-5, pyramidal slip was more activated at low strain rates than at high strain rates.
- For Ti-210 and Ti-30, the work-hardening rate varied as an S-shaped curve. This corresponded to the introduction of twinning.
- In Ti-210, the greater fracture strain and work-hardening ability at higher strain rates were because of the higher frequency of twinning. In Ti-5, wherein the frequency of twinning was lower, the higher fracture strain at lower strain rates was attributed to the activity of the 1st order pyramidal slip. In Ti-30, the fracture strain was constant regardless of the strain rate. This is because of the contribution of twinning at high strain rates and pyramidal slip at low strain rates.

## Figures and Tables

**Table 1:** Chemical compositions of the two pure titanium sheets used in this study (mass%).

Ti	Fe	O	N	C	H
Bal.	0.03	0.03	0.002	0.006	0.0006
Bal.	0.03	0.05	0.01	0.01	0.002

**Table 2:** Mechanical properties obtained from tensile tests.

Specimen	$\dot{\varepsilon }$ [s^−1^]		$\sigma_{0.2}\;[\mathrm{MPa}]$	$\sigma_{UTS}\;[\mathrm{MPa}]$	$\varepsilon_{f}\;[\%]$
Ti-210	10^−6^	Test-1	93	201	39
		Test-2	100	221	47
	10^−4^	Test-1	108	269	45
		Test-2	106	270	46
	10^−2^	Test-1	120	326	55
		Test-2	118	319	56
	10^0^	Test-1	126	354	58
		Test-2	134	350	59
Ti-30	10^−6^	Test-1	118	247	60
		Test-2	120	246	52
	10^−4^	Test-1	127	280	60
		Test-2	147	301	57
	10^−2^	Test-1	148	317	63
		Test-2	152	330	63
	10^0^	Test-1	177	363	63
		Test-2	167	361	65
Ti-5	10^−6^	Test-1	197	369	47
		Test-2	218	379	42
	10^−4^	Test-1	254	395	48
		Test-2	250	402	48
	10^−2^	Test-1	316	413	40
		Test-2	290	415	39
	10^0^	Test-1	325	433	41
		Test-2	315	434	39

**Table 3:** Strain rate sensitivities of $\sigma_{0.2}$ and $\sigma_{\mathrm{UTS}}$.

Specimen	$m\left(\sigma_{0.2}\right)$	$m\left(\sigma_{UTS}\right)$
Ti-210	0.019	0.035
Ti-30	0.033	0.031
Ti-5	0.036	0.011

## Data Availability

The raw/processed data required to reproduce these findings cannot be shared at this time as the data also forms part of an ongoing study.
